# Emerging Applications of Silica Nanoparticles as Multifunctional Modifiers for High Performance Polyester Composites

**DOI:** 10.3390/nano11112810

**Published:** 2021-10-22

**Authors:** Tian Hao, Yao Wang, Zhipeng Liu, Jie Li, Liangang Shan, Wenchao Wang, Jixian Liu, Jianguo Tang

**Affiliations:** 1 National Center of International Research for Hybrid Materials Technology, Institute of Hybrid Materials, National Base of International Science & Technology Cooperation, Qingdao University, Qingdao 266071, China; haotian_678@163.com (T.H.); qduliuzhipeng@sina.com (Z.L.); lijieqdu@163.com (J.L.); shanliangangqdu@163.com (L.S.); wangwenchao1021@163.com (W.W.); 2 College of Materials Science and Engineering, Qingdao University, Qingdao 266071, China

**Keywords:** silica nanoparticles, polyester, nanocomposites, surface modification, fluorescent properties

## Abstract

Nano-modification of polyester has become a research hotspot due to the growing demand for high-performance polyester. As a functional carrier, silica nanoparticles show large potential in improving crystalline properties, enhancing strength of polyester, and fabricating fluorescent polyester. Herein, we briefly traced the latest literature on synthesis of silica modifiers and the resultant polyester nanocomposites and presented a review. Firstly, we investigated synthesis approaches of silica nanoparticles for modifying polyester including sol-gel and reverse microemulsion technology, and their surface modification methods such as grafting silane coupling agent or polymer. Then, we summarized processing technics of silica-polyester nanocomposites, like physical blending, sol-gel processes, and in situ polymerization. Finally, we explored the application of silica nanoparticles in improving crystalline, mechanical, and fluorescent properties of composite materials. We hope the work provides a guideline for the readers working in the fields of silica nanoparticles as well as modifying polyester.

## 1. Introduction

Polyester is the most widely used material in textiles, packaging, engineering plastics, and other fields due to its excellent performance such as high hardness, low density, and good transparency [1,2,3,4]. Nano-modification of polyester has become a research hotspot due to growing demand for high-performance polyester [5,6,7,8]. To date, many nano additives are used to modify polyester, such as carbon fiber [9,10], graphene [11,12], silica [13], etc. However, silica nanoparticles (SNs) are far superior to other materials as modifiers of polyester on account of their high transparency, low density, low cost, and easy availability [14,15,16]. The transparency, mechanical properties, and crystallization properties of the polyester can be greatly enhanced by introducing SNs as functional carriers without side effect [17], or endowed with other functional characteristics like fluorescence [18].

For modifying polyester, size and morphology of SNs are two key parameters, which can be finely controlled by synthesis technologies [19,20,21], including chemical vapor deposition [22,23,24], flame synthesis [25,26], hydrothermal synthesis [27], reverse microemulsion technology [28,29], and sol-gel method [30,31], etc. Recently, sol-gel and reverse microemulsion have been the most widely used approaches for the synthesis of SNs. In 1968, Stöber et al. [32] first synthesized highly monodisperse SNs by sol-gel process, controlling the growth of spherical SNs with uniform size in the range of 0.05–2 μm diameter, which was called the Stöber method. Subsequently, many related studies on synthesis of SNs were reported evolving from the Stöber method [33,34]. Bari et al. [35] prepared SNs with particle sizes of ca. 100 nm to 2 μm by sol-gel method. Several researchers adjusted the size and structure of SNs via sol-gel method by changing the catalyst [36,37], pH [38,39]. Another popular approach for synthesizing SNs is reverse microemulsion method, which was first proposed by Schulman in 1943 [40]. In 1990, monodispersed SNs were prepared by Osseo-Asare and Arriagada by controlling the hydrolysis of tetraethyl orthosilicate (TEOS) via reverse microemulsion [41]. Reverse microemulsion method was used to synthesize silica-based nanocomposites with different structures, and these structures were of decisive significance for their properties and applications [42]. Moreover reverse microemulsion method was mainly used to synthesize mesoporous SNs [43,44,45], hollow SNs [45,46,47], and core–shell SNs [48,49,50,51,52] due to its convenient control on nanoparticle shape.

Before compositing with polyester, SNs are generally surface modified to improve their dispersity in polyester matrix because they are hydrophilic and easy to agglomerate [26,53,54]. There are large amounts of hydroxyl groups on the outlayer of SNs, which provide good chemical reaction sites for the surface modification of SNs [55,56]. In recent years, modification methods of SNs have been widely investigated, including coupling agent modification, polymer modification, etc. [57,58]. Jitjaicham et al. [59] modified fumed SNs with vinyltriethoxysilane, which reduced the agglomeration of fumed SNs and improved the dispersion of fumed SNs in poly(ethylene terephthalate) (PET) fibers. Recently, Kim et al. [60] grafted a new styrylsilane coupling agent on the surface of SNs. Functionalized styrylsilanes, which were readily prepared via catalytic hydrosilylation of the corresponding phenylacetylenes with silanes, were immobilized on SNs through acid catalyzed processes under mild conditions.

To obtain high-performance SNs/polyester composites, material composite methods are crucial even though the dispersibility of SNs was greatly improved after surface modification [61,62,63]. The blending methods of SNs with polyester matrix include physical blending, sol-gel processes, in situ polymerization, etc. Courtat et al. [64] added 10 wt% SNs to dry polybutylene terephthalate (PBT) at 240 °C for melt blending using a co-rotating twin-screw extruder. Polyhydroxybutyrate (PHB)/poly(ε-caprolactone) (PCL)/sol-gel derived silica hybrid scaffolds with a 5:1 organic/inorganic ratio were fabricated through a combination of electrospinning and sol-gel method. The results showed that SNs can greatly enhance the stiffness and strength of PHB/PCL fibers [65]. Achilias et al. [66] prepared PET nanocomposites containing 1 or 2.5 wt% mesoporous SNs (average pore diameter of 4.7 or 14.2 nm) by in situ polymerization. The above several mixing methods all make the SNs disperse and mix in the polyester matrix well despite their own merits.

Nanocomposite technologies are effective methods to improve the physical properties of polymers [67]. The presence of functional groups on the particle surface influences the filler–filler and filler–polyester interactions, and consequently modifies the reinforcement level of the composites [68]. Therefore, the existence of fillers’ perfect polyester crystallinity and mechanical strength and other properties [69,70]. In general, clay [71,72,73], carbon nanotubes [74,75,76], graphene [77], zinc powder [78], and silica [79,80,81] were widely used as nano-fillers to improve the crystallization and mechanical properties of polymer substrates. SNs are rising stars as modifiers of polyester on account of their intrinsic characteristics of light transmission, low density, low cost, and easy availability. Recently, research has been relatively extensive on the effect of silica additives on polyester properties. Ma et al. [82] proved that SNs were effective nucleating agents, which were conducive to the crystallization of PET. With the increase of silicon content, the crystallization speed of PET was obviously accelerated. Chen et al. [83] successfully prepared toughened polylactic acid (PLA) nanocomposites with high stiffness and high rigidity by combination of 5% SNs modification and uniaxial pre-stretching. At present, polyester materials are widely used, and the requirements for polyester materials are not only to improve their own performance, but more importantly, to develop and apply new functions [84,85]. Luminescent polyester materials were considered to be one of the hotspots in functional polyester research [86]. Luminescent polyester material is now a new type of non-toxic, harmless, and non-radioactive environmentally friendly organic polymer material, which is widely used in toys, biology, night operations, fire emergency, and textiles, etc. [87,88,89].

In recent years, silica-based nanocomposites have been reviewed in detail by several researchers in their synthesis methods, properties, and applications [90,91,92,93]. Linhares et al. [93] reviewed silica aerogel composites with embedded fibers, covering the synthesis and properties of silica aerogel and the impact of different fiber embeddings on the final properties of the composite, namely morphology, orientation, and optical features. Saraswathi et al. [91] reviewed the performance of polymeric ultrafiltration membranes incorporating silica-based nanomaterials. Yan et al. [94] reviewed the latest research progress of optically functional rare earth hybrid materials based on polymers and SNs/polymer composite materials. It can be seen that silica-based nanocomposites have made important research progress and have been widely used in practice. However, these reviews mainly introduced SNs/polymer composites and did not focus on fabrication and application of SNs/polyester composites.

In this review, we briefly traced the recent literature on the synthesis and modification of SNs and their effect on the properties of SNs/polyester nanocomposites. First, we surveyed the synthesis and surface modification methods of SNs for modifying polyester. Then, we summarized three compositing methods of SNs/polyester including sol-gel method, blending method, and in situ polymerization. Furthermore, we introduced the application of SNs in improving crystalline, mechanical, and fluorescent properties of composite materials. Additionally, the latest progress in the development and application of fluorescent polyester is also described. We expect that this review provides guidance and assistance to researchers engaged in the polymer composite field.

## 2. Synthesis and Surface Modification of SNs

### 2.1. Synthesis of SNs

Size and structure are the key parameters determining the performance of the fabricated SNs/polyester composites, which should be finely controlled in the synthesis [95]. The approaches for the synthesis of SNs include chemical vapor deposition [23,96,97], flame synthesis [25,26], sol-gel method [98], and reverse microemulsion technology [99], etc. SNs synthesized by chemical vapor deposition have high purity and few surface hydroxyl groups, so they have excellent reinforcement performance and are mostly used for reinforcement of silicone rubber [100]. Flame method is the high temperature flame decomposition of metal-organic precursors for commercial production of SNs in powder form [25]. However, SNs synthesized by the above two methods have serious agglomeration and cannot control the particle size. For the particles used in the polyester composites, sol-gel method and reverse microemulsion are the better choices, performing well in controlling particle morphology and particle size by changing reaction conditions. A better understanding of synthesis methods will provide the basis for the effective application of SNs in polyester composites. The two routines will be introduced in detail as follows.

#### 2.1.1. Sol-Gel Method

Sol-gel method is widely used in synthesis of SNs to get pure and homogeneous products under mild conditions [101,102,103]. The process involves initial hydrolysis and subsequent condensation of silicates employing mineral acids or bases as catalysts. First, silicate is hydrolyzed in a mixture of ethanol, water, and acid or alkali to form silicic acid. Then, condensation/polymerization between silanol groups or between silanol groups and ethoxy groups forms a siloxane bridge (Si-O-Si), thereby forming silica structure. Particle size and morphology of SNs could be controlled by altering catalyst type (acid or base) [37,104], pH [38,39], and the type of solvent [105,106].

**Figure 1:**
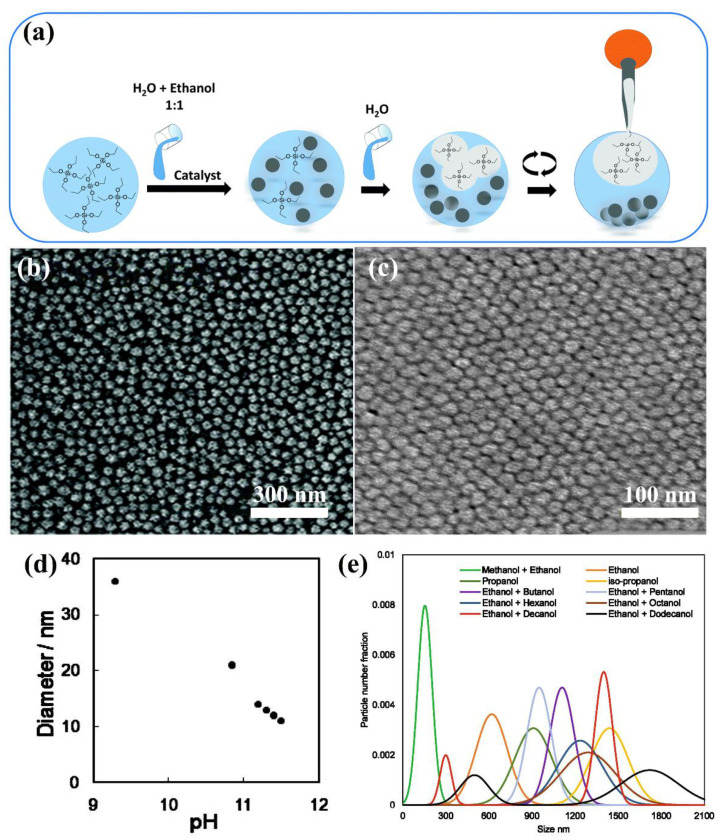
Illustration of sol-gel synthesis of SNs. (**a**) Schematic illustration of the formation process of SNs. Adapted with the permission from [107]. Copyright 2016, Royal Society of Chemistry. (**b**) SEM image of the synthesized SNs at 20 °C for 20 min. Adapted with the permission from [107]. Copyright 2016, Royal Society of Chemistry. (**c**) SEM Image of the synthesized SNs under the condition of pH = 10.8. Adapted from [104]. Copyright 2011, American Chemical Society. (**d**) Relationship between the initial pH of the water phase and the average diameter of SNs. Adapted with the permission from [104]. Copyright 2011, American Chemical Society. (**e**) Particle size distribution of SNs synthesized in different solvents with NH_3_ = 1 M, H_2_O = 7 M, temperature 55 °C. Adapted with the permission from [35]. Copyright 2020, Elsevier.

Dixi et al. [107] synthesized SNs with the controlled particle size via sol-gel method. In the procedure (Figure 1a), TEOS was employed as precursor, an equal volume of ethanol–water mixture as medium and sodium hydroxide as the catalyst. After 20 min of reaction under 20 °C, monodispersed SNs of 50 nm were synthesized (Figure 1b). In the synthesis, the unreacted TEOS and polysilicic acid chains were eliminated by emulsification using supersaturated water, during which TEOS was transferred to the emulsion phase from the solution phase terminating particle growth. Based on this mechanism, particle size could be finely controlled in the sol-gel process. Similarly, Yang and coworkers prepared SNs of 6.4 nm using L-lysine as catalyst via sol-gel process [108].

Wang et al. [104] reported the synthesis of uniform silica nanospheres with low polydispersity (<12%) in a liquid–liquid biphasic system containing TEOS, water, and primary amine (or-ammonia) by controlling pH conditions. In this process, the final size of SNs was adjusted by changing the initial pH of the water phase. Figure 1c shows an SEM image of monodisperse SNs with an average diameter of 21 nm under pH = 10.8. Moreover, the larger the initial pH value of the reaction solution, the smaller the size of the synthesized SNs (Figure 1d).

Bari et al. [35] studied the influence of various alcoholic solvents on the size of SNs synthesized by hydrolysis and condensation process of TEOS. It can be seen in Figure 1e that SNs of size between 100 nm and 2 µm were obtained by changing the solvent composition.

Some researchers further modified the sol-gel process by introducing surfactants or different precursors varieties in the reaction system. Singh et al. [109] confirmed that dodecytrimethylammonium bromide (140 nm), tetradecyltrimethylammonium bromide (95 nm), and cetyltrimethylammonium bromide (55 nm) were used as cationic surfactants to control the particle size of SNs. Kurdyukov et al. [34] reported that the size of SNs was minimized from 50 to 10 nm through addition of [3-(methacryloyloxy) propyl]trimethoxysilane to the silica precursor. Najafi et al. [110] synthesized spherical SNs with an ultrafine particle size of 25 nm via sol-gel method using ammonium polycarboxylate surfactants.

In the sol-gel process, alkyl silanes are gradually hydrolyzed, then condensed with alkoxy groups. Since hydrolysis and condensation reactions are initiated at multiple sites, the reaction kinetics is complex. At a certain point, a three-dimensional network could be formed to get particles. The size and morphology of SNs can be controlled by adjusting reaction parameters like precursor type, pH, catalyst, etc. In general, the particle size of SNs synthesized through sol-gel method could be accurately controlled from a few nanometers to microns with good uniformity and dispersity.

#### 2.1.2. Reverse Microemulsion Method

In the past decade, reverse microemulsion technology was adopted to synthesize SNs [111,112]. In the process, monodisperse water droplets were surrounded by the monolayer interface composed of surfactants and co-surfactants and serve as nano reactors [113,114]. Dispersity, morphology, and size of the synthesized SNs could be effectively controlled in the reverse microemulsion process [115].

**Figure 2:**
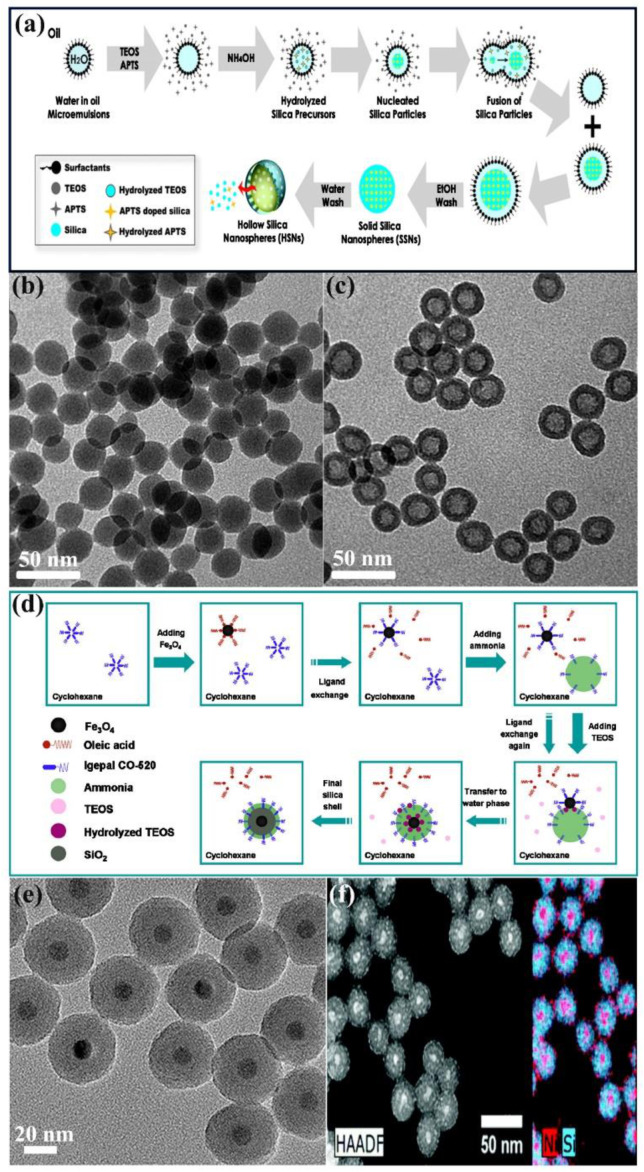
Synthesis of SNs via reverse microemulsion method. (**a**) Schematic diagram of the nucleation and growth mechanism of HSNs. Adapted with permission from [46]. Copyright 2015, Royal Society of Chemistry. (**b**) TEM images of unetched solid SNs and (**c**) TEM images of HSNs. Adapted with permission from [46]. Copyright 2015, Royal Society of Chemistry. (**d**) Schematic diagram of the mechanism coating SiO_2_ on the surface of Fe_3_O_4_ nanoparticles. Adapted with permission from [49]. Copyright 2012, American Chemical Society. (**e**) TEM image of Fe_3_O_4_@SiO_2_ nanoparticles. Adapted with permission from [49]. Copyright 2012, American Chemical Society. (**f**) HAADF-STEM images and EDS maps showing Ni and Si of SiO_2_-Ni NPs. Adapted with permission from [52]. Copyright 2017, Royal Society of Chemistry.

Lin et al. [46] fabricated hollow silica nanospheres (HSNs) via reverse microemulsion method. In the reaction system containing ammonia, TEOS, and aminopropyltrimethoxysilane (APTS), reverse micelles were employed as soft containers to initiate the nucleation and growth of SNs forming HSNs. As illustrated in Figure 2a, the condensation of TEOS and APTS in the core region was incomplete leading to HSNs with interior hollow cavity and nano porous shell after etching in warm water. Figure 2b,c shows representative TEM images of unetched solid SNs and HSNs. Moreover, the oil phase of alkanes with different alkyl chains was systematically investigated to tune the size of HSNs by changing the molar ratio of water-in-oil (W/O), the amount of co-solvent and surfactants. Similarly, the HSNs were synthesized using a modified W/O reverse microemulsion system (containing APS, Triton X-100, n-hexanol, cyclohexane, and water), and hollow mesoporous silica nanospheres (HMSNs) were also prepared by adding the pore-generating reagent (cetyltrimethyl-ammonium bromide) in the reaction mixture. The prepared HSNs and HMSNs with the size less than 50 nm were spherical uniform, and the core size and cavity volume of the particle could be tunable [47].

Reverse microemulsion was frequently used to deposit SiO_2_ shells on pre-synthesized inorganic nanoparticles to form core/shell nanoparticles [45,52,116,117]. Ding et al. [49] proposed to synthesize Fe_3_O_4_@SiO_2_ core/shell nanoparticles by reverse microemulsion method. Figure 2d shows a schematic diagram of the coating mechanism of SiO_2_ on the surface of Fe_3_O_4_ nanoparticles. First, Igepal CO-520 aggregates and forms micelles due to its hydrophilic groups in cyclohexane solution. Second, when Fe_3_O_4_ nanoparticles are added to the solution, ligand exchange occurs between oleic acid and part of Igepal CO-520 on the surface of Fe_3_O_4_ nanoparticles. Then, ammonia is added to fill the remaining Igepal CO-520 micelles, and the size of micelles will be enlarged and form a reverse microemulsion system. Subsequently, the added TEOS will be hydrolyzed at the oil/water interface and undergo ligand exchange with Fe_3_O_4_ nanoparticles surface, and then it will be transferred to the water phase. Finally, TEOS undergoes a condensation process and forms silica shells to obtain Fe_3_O_4_@SiO_2_ core/shell nanoparticles. Additionally, in this process, by adjusting the parameters (such as the content of Fe_3_O_4_ nanoparticles, Igepal CO-520, ammonia, and TEOS), Fe_3_O_4_@SiO_2_ nanoparticles with different shell thickness can be obtained. Figure 2e illustrates TEM image of Fe_3_O_4_@SiO_2_ nanoparticles, in which a spherical core/shell structure with just 12.2 nm single-core Fe_3_O_4_ nanoparticles and a 14.9 nm shell thickness SiO_2_ coating can be seen. Similarly, Lynch et al. [52] reported that SiO_2_ was deposited on Ni nanoparticles by the reverse microemulsion method to form SiO_2_-overcoated Ni nanoparticles (SiO_2_-Ni NPs) with an average diameter of 30 nm. According to the High-angle Annular Dark-field Scanning Transmission Electron Microscopy (HAADF-STEM) and Energy Dispersive Spectroscopy (EDS) diagram of SiO_2_-Ni NPs, each SiO_2_-Ni NPs contained a ~7 nm oxidized Ni core, and a large number of oxidized Ni NPs with diameters of 2 nm were distributed throughout the SiO_2_ shell (Figure 2f). Dahlberg et al. [50] synthesized SiO_2_ nanotubes containing Ni nanoparticles in a templated nonionic surfactant W/O microemulsion. Almana et al. [51] also synthesized Pt@SiO_2_ core-shell nanoparticles via a water-in-oil reverse microemulsion. This kind of core-shell structured nanoparticles not only has the surface properties of SNs, but also can be multifunctional by combining other types of materials (such as quantum dots or magnetic nanoparticles [49]). Moreover, the particle properties and shell surface chemistry can be adjusted by controlling the core size or shell thickness [117,118].

Reverse microemulsion approach has shown large potential in synthesizing SNs with the rationally designed particle size and morphology because the reaction proceeds in reverse micelles of controllable size and good dispersibility. Additionally, lots of nanoparticles with abnormal shape or heterostructure can be fabricated using reverse microemulsion technology. However, its applications are largely limited by high cost and difficulty in removing surfactant, especially for large-scale production.

### 2.2. Surface Modification of SNs

The surface of SNs is hydrophilic, leading to poor compatibility and easy agglomeration when it is combined with organic polymer matrix [119]. A large amount of attention has been attracted to modifying physical and chemical properties of the SNs surface to enhance the compatibility between SNs and PET and processability of the composites [120,121,122]. The better routines are grafting silane coupling agent modification and polymer onto the surface of SNs particles, which are demonstrated as following [123].

#### 2.2.1. Grafting Silane Coupling Agent

Silane coupling agents are the most commonly used modifiers for modifying SNs [124]. Silane coupling agents have dual reactive functional groups, and its general formula is usually expressed as RSiX_3_. In the formula, X is a hydrolyzable group, such as chlorine, methoxy, ethoxy, isopropyl functional group, which can be hydrolyzed and condense with the active hydroxyl group on the surface of the nanoparticle to form a siloxane bond, thereby connecting to the surface of the nanoparticle. Additionally, R stands for alkyl chains that are compatible with polyester. Table 1 lists some typical silane coupling agents used for surface modification of SNs.

**Figure 3:**
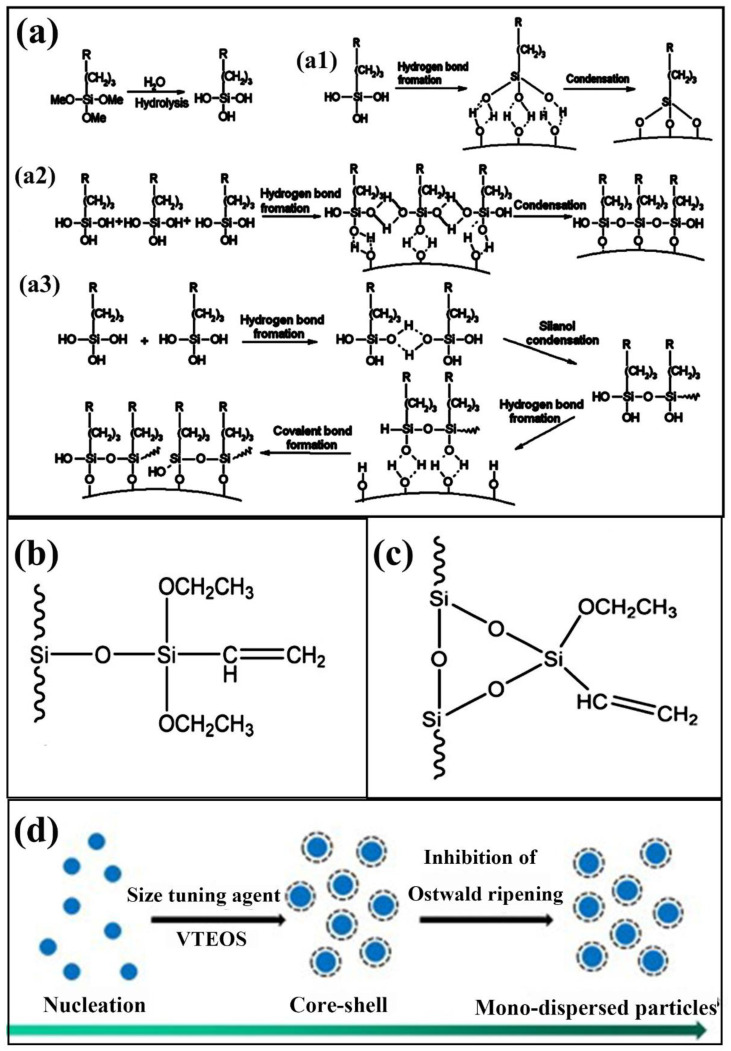
Legend of silane coupling agent grafting SNs. (**a**) Three possible mechanisms for grafting reaction between MPS and SNs (**a1**–**a3**). Adapted with permission from [26]. Copyright 2015, Elsevier. (**b**,**c**) Two connection types of coupling agent VTEOS. Adapted with permission from [132]. Copyright 2021, Royal Society of Chemistry. (**d**) Schematic diagram of VTEOS size adjustment mechanism. Adapted with permission from [133]. Copyright 2012, Elsevier.

Zhang et al. [26] successfully converted hydrophilic SNs to hydrophobic ones through silylation reaction between γ-methacryloyloxypropyltrimethoxysilane (MPS) and hydroxyl groups on the SNs surface. MPS molecules were grafted to the SNs surface through three mechanisms (Figure 3a). In the beginning, MPS was hydrolyzed in the presence of water and acid to produce reactive Si-OH groups. Subsequently three possibilities exist: first, hydrolyzed MPS molecules may form three hydrogen bonds with three adjacent Si-OH groups on SNs and then undergo a condensation reaction (Figure 3(a1)). Second, MPS molecules may react with Si-OH of the adjacent MPS molecules (Figure 3(a2)). Thirdly, the MPS molecules condense with each other, resulting in a chain or network structure grafted to the SNs surface (Figure 3(a3)). Wang et al. [132] studied the grafting modification mechanism of the coupling agent vinyltriethoxysilane (VTEOS) to macroporous silica gel. The coupling agent molecules have two types of connection on the surface of silica gel (Figure 3b,c), and the proportions of these two types are 43.51% and 56.49%, respectively. The average grafting rate of coupling agent VTEOS on macroporous silica gel was 91.03% under optimized conditions. Lin et al. [133] prepared monodisperse core-shell silica hybrid spheres using VTEOS as capping and size tuning agents. The schematic diagram of the VTEOS size tuning mechanism (Figure 3d) showed the formation of a vinyl organic shell on the surface of silica core. Pandis et al. [126] synthesized PLA/silica membranes. Silica was synthesized by ammonia decomposing with TEOS and 3-glycidyloxypropyltrimethoxysilane (GPTMS) as precursors, which entered into the pores of the PLA membrane by sol-gel reaction in situ. Lai et al. [129] grafted thermoplastic polyurethane onto the surface of SNs with (3-aminopropyl)triethoxysilane (APTES) as the coupling agent, which acted as the nucleating agent to blend with PLA to improve the crystallization performance of PLA. It was proved that the optical transmittance of modified silica composite was higher than that of unmodified silica composite.

#### 2.2.2. Grafting Polymer

Grafting polymers onto SNs is another effective way to improve interfacial interactions in nanocomposites [134]. Generally, there are two main approaches to chemically attaching polymer chains to the surface of SNs. One is to covalently graft organic monomers to the surface of SNs (“grafting to” method); the other is to rely on the initiator to grow polymer chains from the surface of SNs through polymerization (“grafting from” method). The “grafting to” method involves connecting pre-formed polymer chains with terminal functional groups (such as, -OH, -Cl, -COOH, etc.) to the surface of SNs through chemical bonds. This method can graft most polymers to the surface of SNs, and the grafted polymer has the advantage of controllable relative molecular weight [135,136,137]. The “grafting from” method is also called surface-initiated polymerization, which refers to the use of the reactive groups on the surface of SNs to bond with the initiator, and then the initiator acts as the active site for surface polymerization to initiate the polymerization of monomers and finally form a polymer [138,139]. Although the experimental process is more complicated, the “grafting from” method can achieve higher grafting rate and is widely used.

**Figure 4:**
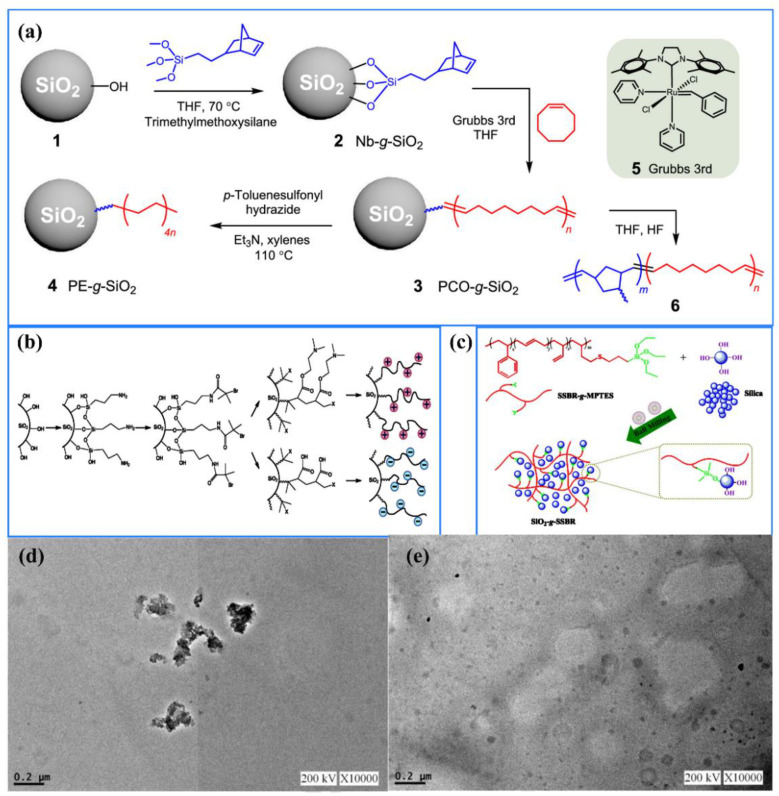
Legend of polymer grafted SNs modification. (**a**) Synthesis flow chart of SNs grafted with poly(cyclooctene) and polyethylene. Adapted with permission from [140]. Copyright 2020, American Chemical Society. (**b**) Schematic diagram of surface modification and polymerization of PDMAEMA or PMAA on SNs. Adapted with permission from [143]. Copyright 2018, American Chemical Society. (**c**) Diagram of SSBR-g-MPTES grafted onto SNs by ball milling. Adapted with permission from [144]. Copyright 2019, American Chemical Society. (**d**) PHB with 5 wt% bare SNs. Adapted with permission from [145]. Copyright 2017, Elsevier. (**e**) PHB with 5 wt% PEG-silica. Adapted with permission from [145]. Copyright 2017, Elsevier.

Zhu et al. [140] synthesized poly(cyclooctene) and polyethylene grafted SNs through fast surface-initiated ring-opening metathesis polymerization by tethering Grubbs third generation catalyst on the surface of particles. Figure 4a shows the synthesis pathway for preparing poly(cyclooctene) and polyethylene grafted SNs. Firstly, SNs were modified with norbornyl trimethoxysilane, and the unreacted hydroxyl groups on the particles were capped with excess trimethylmethoxysilane. Then Grubbs’ third-generation catalyst was bound to a norbornyl group and used as an initiator for cyclooctene monomer in the subsequent steps. Finally, polycyclooctene-grafted SNs were obtained, which were slightly hydrogenated to produce polyethylene-grafted SNs [141]. Dang et al. [142] prepared poly(styrene-r-acrylonitrile) grafted SNs by surface-initiated atom transfer radical polymerization. Sokolowski et al. [143] synthesized polymer brush grafted anionic SiO_2_@poly(methacrylic acid) (PMAA) and cationic SiO_2_@poly(2-(dimethylamino)ethyl methacrylate) (PDMAEMA) inorganic/polymer hybrid nanoparticles through surface-initiated atom transfer radical polymerization, and is depicted in Figure 4b. Wen et al. [136] grafted methoxypolyethylene glycol onto amino-modified SNs (SiO_2_-NH_2_) through terminal groups reaction between epoxide terminated PEG and SiO_2_-NH_2_ in a mixed solution (n-decane/toluene), a “grafting to” routine [137]. Gao et al. [144] reported a simple and efficient method for preparing highly dispersible silica-g-solution styrene butadiene rubber (SSBR) through mechanochemical activation obtained by ball milling (Figure 4c). The condensation reaction between the siloxane group of SSBR-g-3-mercaptopropyltriethoxysilane (MPTES) and the hydroxyl group on the silica surface successfully grafted SSBR-g-MPTES onto the SNs, and the maximum grafting percentage was 6.9%. Lan et al. [145] studied the effect of the surface properties of SNs on the dispersion of SNs in the PHB matrix. Results indicated that PEG-modified SNs (Figure 4e) can significantly improve the dispersibility of SNs in the PHB matrix compared to bare SNs (Figure 4d). Chang et al. [146] grafted acrylic copolymer onto SNs via 3-(trimethoxysilyl) propyl methacrylate and trimethoxysilane, and the obtained polymer/silica hybrid materials can enhance the mechanical strength and thermal stability of PET matrix.

Many new properties are endowed with SNs such as amphiphilicity, organic compatibility, and dispersibility, etc., when polymer or silane coupling agent is chemically bonded to the surface of SNs. Surface modification has become an important research topic in the field of materials science because the as prepared hybrid materials showed excellent performance and large potential in field of composite materials.

## 3. Processing Method of SNs/Polyester Nanocomposites

SNs/polyester nanocomposites have gained great concern due to their excellent performance and large application potential. The dispersion of SNs in polyester matrix is the key parameter determining final performances of the composite materials [147,148]. To date, three methods can achieve good dispersion of fillers in polyester [149,150], including physical blending, sol-gel processes, and in situ polymerization, which will be introduced in detail as follows.

### 3.1. Physical Blending

Physical blending is a simple method for preparing polymer-based nanocomposites by mixing the synthesized nanoparticles with polymers [151,152]. In terms of blending mechanisms, physical blending is divided into solution blending and melt blending. In the process of solution blending, nanoparticles are first added to polyester solution under strong stir forming uniform dispersion. Then, the solvent is removed getting desired SNs/polymer composite. Melt blending of SNs with polymer matrix has been widely used due to its high efficiency, environmentally friendliness, and ease of operation. During the process, SNs are uniformly dispersed in polymer matrix by means of plasticization and mixing effect achieving desired polymer composites.

Russo et al. [153] prepared PBT-based nanocomposites with different content of SNs using trifluoroacetic acid as a solvent through a solution blending method. SEM images revealed that 3.0 wt% SNs can disperse well in the polymer matrix. It was also proved that the dynamic mechanical properties of nanocomposites with SNs were significantly improved, and the glass transition temperature of nanocomposites with SNs moved to a higher temperature with the increase of SNs content. Hajiraissi et al. [154] melt-blended PBT particles and SNs at a mixing temperature of 240 °C. After blending, the SNs surface groups had a certain interfacial interaction with the carboxyl or hydroxyl end groups of PBT chain. Lai et al. [155] reported the effect of polyethylene glycol methyl ether (PEGME) modified SNs on the properties of PLA by melt mixing. Qi et al. [156] incorporated surface-treated SiO_2_ aerogel nanoparticles into the PBT matrix by melt blending, and presented as independent clusters composed of tens of individual nanoparticles, which were randomly and uniformly distributed in the matrix.

### 3.2. Sol-Gel Processes

The sol-gel process goes beyond the traditional blending method since it can control the morphology or surface characteristics of the inorganic phase in the polymer matrix employing some specific technique [157]. For example, common process usually produces opaque composites with phase sizes much larger than 100 nm, and more generally in the micron range. However, in the sol-gel process adopting acid catalysis, transparent nanocomposites with characteristic morphological dimensions less than 100 nm are usually obtained [158,159].

Mazraeh-shahi et al. [160] prepared a silica aerogel/PET nonwoven fiber composite with excellent performance via a two-step sol-gel process. The synthesized silica aerogel particles completely filled the micron-level pores of PET nonwoven fiber, realizing efficient silica aerogel coating. Su et al. [161] proposed a simple vapor–liquid sol-gel method for manufacturing superhydrophobic polydimethylsiloxane@silica surface on PET textiles. The PET textile was dipped into a mixture of polydimethylsiloxane (PDMS) and tetraethyl orthosilicate, and then equilibrated for 60 min in a reaction chamber filled with 20 wt% HCl vapor at a certain temperature. Finally, the superhydrophobic PDMS@silica surface on PET textile was obtained by drying and removing the generated water in the condensation reaction process in an oven.

### 3.3. In Situ Polymerization Processes

In the insitu polymerization process, nanoparticles are first dispersed in polymer monomers, and then the monomers are polymerized forming uniform composite [162,163]. Generally, the in situ polymerization process involves three consecutive steps [164]. Firstly, nano-additive is pretreated with appropriate surface modifier. Then, modified additive is dispersed into monomer for bulk polymerization or solution polymerization. Finally, nanocomposites are formed during the polymerization process.

Ramamoorthy et al. [165] in situ synthesized silica aerogel/PET nonwoven composites with various silica content, and the average mesopore size of silica aerogel was about 62 nm. Lu et al. [166] prepared PET/SNs hybrid composite by in situ polycondensation of terephthalic acid and ethylene glycol in the presence of SNs pretreated with a silane coupling agent. This polymerization process ensures that the SNs or fine agglomerates were well dispersed in the PET matrix with a size range of 40–60 nm. Additionally, SNs as a heterogeneous nucleating agent effectively improved the crystallization behavior of PET nanocomposites.

In summary, physical blending is simple and easy to implement and industrialize, compared with in situ polymerization and sol-gel process. Among them, solution blending requires a large amount of organic solvent like toluene, which is non ecofriendly. On the contrary, melt blending does not require any solvent, which is more conducive to industrial production. Compared with the traditional blending approach, processing condition of sol-gel method is relatively mild and the dispersion of SNs in polymer is more uniform endowing products with better performances. In situ polymerization is a simple process, during which polymerization of the monomer and composition of material parts are completed simultaneously. Generally, composite prepared via in situ polymerization shows SNs high uniformity, and the one-time polymerization-modeling process ensuring stability of SNs/polyester nanocomposites.

## 4. Application of SNs/Polyester Composites

The properties of SNs/polyester nanocomposites mainly depend on the composition of the inorganic phase, particle size and degree of dispersion [167,168]. The introduction of SNs into polyester can largely improve crystallization properties, mechanical properties and other properties of the nanocomposites [69]. Moreover, the good optical transparency of SNs can be used to obtain fluorescent PETs, further extending application potential of SNs/polyester composites [169].

### 4.1. Enhancing Crystalline Properties

The impurities in polyester have a great influence on its crystallization process [170]. SNs acts as a crystal nucleus in the crystallization process of polyester as an impurity promoting crystallization. The addition of SNs enhances the amount of heterogeneous nucleation, promotes the crystallization of molecules, and accelerates crystallization speed [171]. In recent years, in order to improve the nucleation effect of SNs, pretreatment of SNs has been carried out, which gives its end groups a specific polarity, thereby improving its dispersibility and nucleation effect in polyester [136,172].

**Figure 5:**
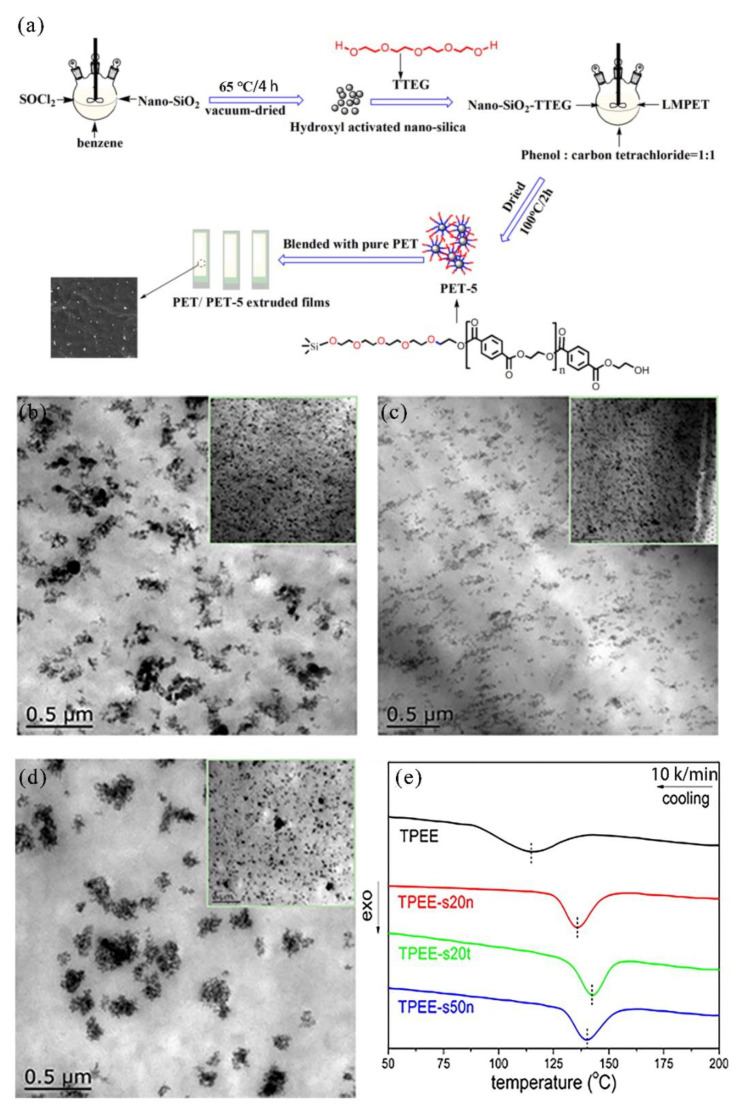
Investigation of the influence of SNs on the crystallization properties of polyester. (**a**) Scheme of interaction mechanism between PET and PET-5. Adapted with permission from [174]. Copyright 2018, MDPI. TEM images of (**b**) TPEE-s20n, (**c**) TPEE-s20t, and (**d**) TPEE-s50n. (**e**) DSC thermograms of neat TPEE and its nucleated samples obtained at a cooling rate of 10 °C·min^−1^. Adapted with permission from [175]. Copyright 2016, American Chemical Society.

Xu et al. [173] studied the effect of SNs on the crystallization behavior of PET. For non-isothermal crystallization and isothermal crystallization, the crystallization rate of PET increased significantly with the increase of SiO_2_ content, but the relative crystallinity of nanocomposites is not different from that of pristine PET. Han et al. [174] prepared three novel nucleating agents, SiO_2_-diethylene glycol-LMPET (PET-3), SiO_2_-triethylene glycol-LMPET (PET-4), and SiO_2_-tetraethylene glycol-LMPET (PET-5), by grafting different oligomers on the surface of SNs and then linking low molecular weight polyethylene terephthalate (LMPET) to enhance the crystallization properties of polyethylene terephthalate (PET). The mechanism of the above whole reaction process is shown in Figure 5a. Differential scanning calorimetry (DSC) study of the composite material of pure PET blended with PET-3, PET-4, and PET-5 showed that the longer ethoxy segment in the nucleating agent showed higher crystallinity, faster crystallization speed, and higher crystallization temperature. For PET-5, the maximum increase for the crystallization peak temperature ranged from 199 to 216 °C, and crystallinity ranged from 23% to 30% at a cooling rate of 10 °C·min^−1^. In conclusion, grafted SNs improved the crystallization properties of PET.

Chen et al. [175] studied the crystallization behavior of thermoplastic PET elastomer (TPEE) with SNs as nucleating agents. Three kinds of SNs with different sizes (20 and 50 nm) and surface treatments with KH-570 were investigated as nucleation agents. The results showed that SNs could be well dispersed in TPEE, but their dispersion and distribution state strongly depended on the particle size and surface treatment. As shown in Figure 5b–d, all three types of SNs were completely distributed throughout the matrix. However, their dispersion state was different. The pristine SNs were dispersed as small aggregates in the TPEE matrix, and the size distribution was broadened with increasing particle size. As can be seen from Figure 5e, the crystallization temperature (Tc) of all samples filled with SNs is about 20–30 °C higher than pure TPEE, which indicates that SNs acts as additional surface nucleation sites, promoting nucleation in crystallization process of matrix TPEE.

Hong et al. [131] analyzed the crystallization behavior of PHB filled with hydrophobic SNs, which were realized through surface modification by methyl methacrylate and amidoamine (AMDA), respectively. It also indicated that AMDA-modified hydrophobic SNs can better increase the crystallization rate of PHB than unmodified hydrophilic SNs. Sarikhani et al. [176] studied the crystallization behavior of PLA with different SNs contents under isothermal and non-isothermal conditions. The crystallization rate increased with the increase of silica loading, and the nanocomposite showed a higher crystallization rate at higher temperatures.

### 4.2. Strengthening Mechanical Properties

The key to strengthening or toughening polyester lies in the dispersion and the interface interaction between SNs and polyester matrix [177]. Surface modification of SNs is used to improve interfacial compatibility with the matrix and its dispersion in the matrix, which is an important factor to strengthen and toughen the matrix material [178]. Additionally, through chemical bond connection or physical entanglement of SNs, polyester matrix has a good interface bond, thus to achieve the composite material to strengthen and toughen [179].

**Figure 6:**
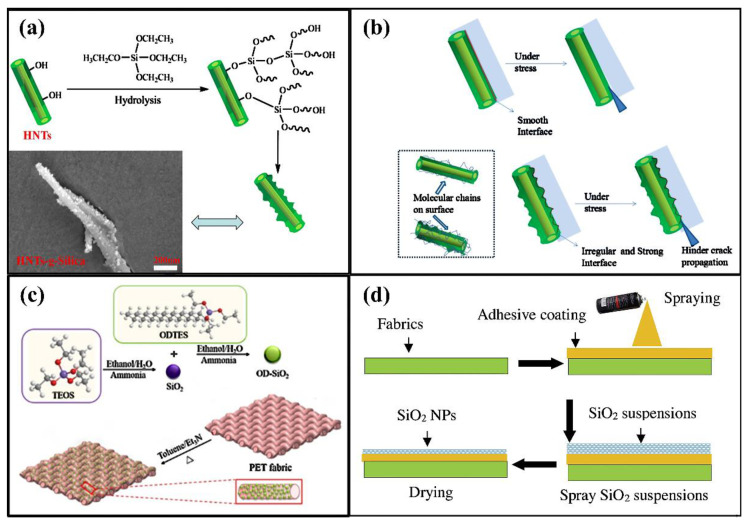
Diagram of the effect of SNs on the mechanical properties of PET. (**a**) Schematic diagram of preparation and morphology of HNTs-g-SiO_2_. Adapted with permission from [79]. Copyright 2017, Elsevier. (**b**) Improved impact strength mechanism model diagram. Adapted with permission from [79]. Copyright 2017, Elsevier. (**c**) Schematic illustration of the preparation of n-octadecyltriethoxysilane modified SNs and coated PET fabric. Adapted with permission from [180]. Copyright 2020, Korean Society of Industrial Engineering Chemistry. (**d**) Method schematic of hydrophobic SNs coated PET fabric. Adapted with permission from [181]. Copyright 2020, Elsevier.

Lin et al. [79] prepared a new type of hybrid nano-filler by in situ growth of SNs on the surface of halloysite nanotubes (HNTs) to improve the impact strength and toughness of unsaturated PET resin (UPE). Figure 6a shows the nanocrystals formed on the surface of HNTs by the Si-O bond uniformly dispersed on the surface of HNT, and the structure and morphology of HNTs-g-Silica. As the specific surface area of HNTs-g-Silica increased, the interfacial interaction between the polymer and HNTs-g-Silica was enhanced. This results in a higher impact strength of UPE composites loaded with HNTs-g-Silica compared with the original HNTs composite, as shown in Figure 6b. It was concluded that HNTs-g-Silica could toughen the unsaturated PET resin.

Kong et al. [180] coated an organic–inorganic hybrid material consisting of n-octadecyltriethoxysilane (OD) modified silica nanoparticles (OD-SNs) onto PET fabrics by a “dipping-drying” approach. This process was illustrated schematically in Figure 6c. Firstly, OD-SNs were synthesized by adding TEOS and OD to the mixed solution of water and ethanol under the condition of ammonium hydroxide as catalyst. Then OD-SNs nanoparticles were dispersed in toluene with triethylamine as catalyst to form a coating solution. Finally, the PET fabric was immersed in the coating solution and dried to get the coated PET fabric. Additionally, experimental tests showed that the excellent mechanical wear resistance of the coated PET fabric was attributed to the coating with OD-SNs. Textiles with a superhydrophobic coating prepared by spraying a commercial adhesive and hydrophobic SNs onto the surface of a PET fabric have good mechanical stability, as shown in Figure 6d [181].

Ramachandran et al. [182] blended different mass fractions (0, 2, 4, 6, 8 wt%) of SNs with PLA to prepare PLA/SNs nanocomposite filament. The mechanical properties of PLA/SNs were studied, and the results showed that the tensile strength, bending strength and impact strength of PLA/SNs all reached the highest value of 52 MPa when the addition amount of SNs was 8 wt%. Weerasunthorn et al. [183] studied the effect of bare fumed SNs and GPMS-modified fumed SNs on the mechanical properties of polybutylene succinate (PBS)/fumed SNs composites. Compared with PBS/fumed SNs composite, PBS/GPMS-modified SNs has better tensile strength, tensile modulus, impact strength, and flexural strength. The optimum loading of GPMS-modified SNs in the PBS was found to be 3 wt%.

### 4.3. Fluorescent Materials

Fluorescent polyester materials refer to the materials that could emit light under irradiation of light of specific wavelength [184]. In recent years, people have begun to pre pare fluorescent materials through compositing organic photosensitive substances and inorganic substrates, in which fluorescent components are uniformly dispersed into base materials [185]. Due to the excellent luminescence properties of rare earth complexes, fluorescent SNs can be synthesized by coating rare earth complexes to SNs, and further blended with polyester matrix achieving fluorescent materials [186].

The research on rare earth polymer composite materials began in the 1960s. Wolf et al. [187] prepared Eu(TTA)_3_ in a ratio of 1:3 between the rare earth ion europium solution and TTA(Tolyltriazole) solution for the first time, and then added Eu(TTA)_3_ into the polymethyl methacrylate matrix. Additionally, they tested the fluorescence properties of the obtained composite materials, opening a new chapter in the research of rare earth polymer materials. Zhang et al. [188] synthesized SiO_2_@Tb^3+^(poly(ethylene terephthalate)-tetraglycol)_3_ phenanthroline (SiO_2_@Tb^3+^(PET-TEG)_3_Phen) as a luminescent nucleating agent for PET substrate. Among them, SNs were used as nucleation sites to improve the crystallization performance; Tb^3+^ played the role of the fluorescence center; the PET-TEG segment acted as a linker and buffer, providing better compatibility with inorganic PET substrates. Therefore, SiO_2_@Tb^3+^(PET-TEG)_3_Phen not only promoted the crystallization rate of PET, but also provided PET with excellent fluorescence properties, which could be used to fabricate fluorescent fibers and textiles.

**Figure 7:**
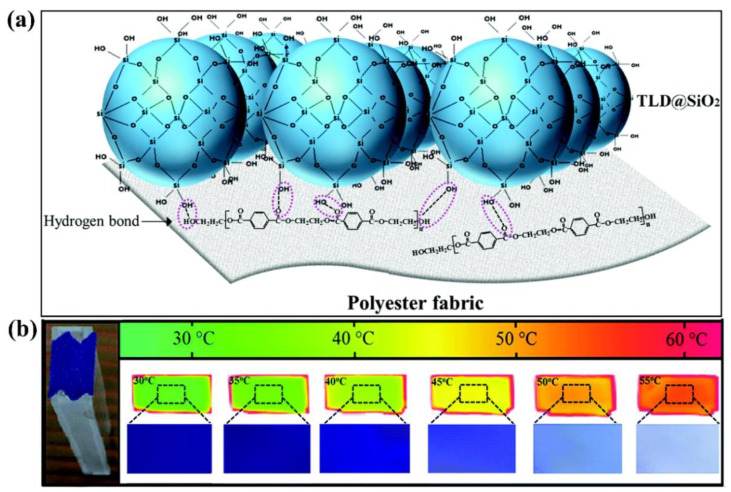
Schematic diagram of fluorescent polyester. (**a**) Schematic illustration of dyeing PET fabric with TLD@SiO_2_. Adapted with permission from [189]. Copyright 2017, Royal Society of Chemistry. (**b**) Reversible color change of thermochromic PET fabric under different temperature. Adapted with permission from [189]. Copyright 2017, Royal Society of Chemistry.

Zhang et al. [189] prepared thermochromic PET fabrics via dyeing with thermochromic leuco dye-loaded silica nanocapsules (TLD@SiO_2_). TLD@SiO_2_ was prepared by hydrolysis of tetraethyl orthosilicate and condensation on the surface of emulsified thermochromic colorless dye (TLD) nano-droplets by sol-gel method. PET fabrics were dyed by immersing them in a dye solution prepared by TLD@SiO_2_ and deionized water in an IR dyeing machine, with pH adjusted to 6. Then, they were heated so that the PET fabric was uniformly dyed. A schematic diagram of PET fabric dyed with TLD@ SiO_2_ is shown in Figure 7a. The resulting PET fabric has thermochromic reversibility, with color reversibly changing from dark blue (25 °C) to light blue (45 °C) and white (80 °C), as shown in Figure 7b. Moreover, thermochromic PET fabrics had excellent colorfastness due to the cross-linked structure of silica nanocapsules among the fibers.

The properties of polyester can be improved effectively when SNs are dispersed into polyester. Firstly, SNs can form new nucleation centers in the polyester matrix, which play a role of heterogeneous nucleation to induce the crystallization of polyester, thus improving the crystallization performance of polyester. Secondly, physical and chemical interaction between SNs and polyester will strength binding force of the components, further strengthening and toughening the composite. Thirdly, SNs could be endowed with different of function by various modification, further mixed with polyester to achieve function materials like fluorescent polyester. The improvement of SNs/polyester nanocomposites broadened the application field of polyester and promoted research and application of nanocomposites.

## 5. Summary and Perspectives

In this work, we reviewed the recent advances in the synthesis and modification of SNs as well as their applications for modifying polyester. In the past few years, a great progress was achieved in improving performance of polyester using SNs as modifiers, especially in enhancing crystallization, mechanical strength of polyester, and fabricating fluorescent composites. In polyester matrix, SNs act as both crystal nucleus and gelation center enhancing crystallization performance and toughness of polyester synchronously. Various functional additives could be made of SNs by grafting and/or conjugating processes, which could be uniformly dispersed in polyester matrix obtaining the composites with the desired function. The dispersion of SNs in the polyester matrix is the most critical factor affecting the final performance of the resultant composites. However, large challenges still stand in the way, which could be concluded as follows.

1. Polydisperse SNs. Although monodisperse SNs could be synthesized by reverse microemulsion method, it is a large issue to remove surfactant used in the process. It is urgent to develop new methods to synthesize small size and monodisperse SNs through ecofriendly processes. Our group has conducted a novel single micelle protocol for synthesizing monodisperse silver nanoparticles and silver sulfide nanoparticles, which should be of large potential in fabrication of monodisperse SNs [190,191,192].
2. Lower grafting ratio of SNs. Grafting ratio is crucial to dispersion of SNs in polyester matrix. However, the grafting rate of silane coupling agent or polymer is both lower than 30% by now. Therefore, it is of great significance to find new modifiers of low steric hindrance or new modifying approaches to improve the grafting rate.
3. Nonuniform composite. The blending process directly affects the dispersity and interfacial interaction between SNs and polyester matrix, which is the key to improve the performance of the composites. Some uniform composites can be prepared by in situ polymerization, but for more materials the uniformity needs to improve by developing efficient blending processes.

Due to high chemical activity of SNs, it can be modified. We can suggest that SNs can be further applied in following fields:

1. Antistatic polyester fiber. SNs can be capped with hydrophilic molecules or antistatic molecules to improve the moisture absorption of SNs/polyester composites.
2. Anti-flaming polyester composite. Capping SNs with flame retardants could uniformly disperse flame retardants together with SNs in polyester matrix getting desired anti-flaming materials.

In summary, SNs are good modifiers for high performance polyester composites and have shown large potential in enhancing mechanical strength of polyester or fabricating new functional polyester composites. We hope this work provides guideline for researchers working in polymer composite fields, and more high-performance polyester composites can be developed enriching our lives.

## Figures and Tables

**Table 1:** Silane-coupling agents used for grafting SNs.

Name	Abbreviation	Chemical Structure	Reference
3-glycidyloxypropyltrimethoxysilane	GPTMS	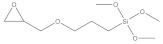	[125,126,127]
(3-aminopropyl) triethoxysilane	APTES	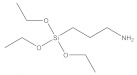	[127,128,129]
(3-Mercaptopropyl) triethoxysilane	MPTS	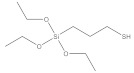	[130]
γ-methacryloyloxypropyltrimethoxysilane	MPS	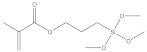	[26,131]
Vinyltriethoxysilane	VTEOS	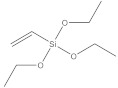	[132,133]

## Data Availability

The study did not report any data.
